# Adherence to the physical activity guideline beyond the recommended minimum weekly amount: impacts on indicators of physical function in older adults

**DOI:** 10.3389/fpubh.2023.1197025

**Published:** 2023-06-13

**Authors:** Jort Veen, Peter Edholm, Lara Rodriguez-Zamora, Mattias Folkesson, Fawzi Kadi, Andreas Nilsson

**Affiliations:** School of Health Sciences, Örebro University, Örebro, Sweden

**Keywords:** exercise, sarcopenia, aging, muscle strength, physical performance, protein intake, six-minute walk test

## Abstract

**Introduction:**

The extent to which additional health benefits of accumulating twice the minimum amount of time in moderate-to-vigorous physical activity (MVPA) affects indicators of physical function in older adults is unclear. Therefore, the aim of the present study was to assess indicators of physical function in older adults who accumulate at least 150 but less than 300 min/week of MVPA compared to those accumulating at least 300 min/week.

**Methods:**

Indicators of physical function, including handgrip strength, 5 times sit-to-stand test (5-STS), squat jump and 6-min walk test (6MWT) were assessed in a sample of 193 older men (*n* = 71, 67 ± 2 years), and women (*n* = 122, 67 ± 2 years), who all accumulated at least 150 weekly minutes of MVPA. Time in MVPA was assessed by accelerometry during 1 week and engagement in muscle strengthening activities (MSA) was assessed by self-report. Protein intake was assessed by a food-frequency-questionnaire. Participants were classified as physically active (≥150 but <300 min of MVPA per week) or as highly physically active (≥300 min of MVPA per week).

**Results:**

Factorial analysis of variance revealed that older adults accumulating at least 300 min of MVPA per week had a significantly (*p* < 0.05) better 6MWT performance and overall physical function compared to the less active group. These findings remained significant after further adjustment for MSA, sex, waist circumference and protein intake. In contrast, no significant differences in indicators of muscle strength were observed between the two groups.

**Discussion:**

Adherence to twice the recommended minimum amount of weekly MVPA time is related to a better physical function, evidenced by a better walking performance compared to adherence to the minimum weekly amount of MVPA. This finding emphasizes the benefits of accumulating daily MVPA beyond the minimum recommended amount to optimize the ability to perform activities of daily living, thus reducing the burden of physical disability and related health-care costs.

## 1. Introduction

The aging process is accompanied by a gradual decline in physical performance including compromised muscle strength, muscle power and aerobic capacity (1–3). These changes lead to a reduced physical function, which in turn may limit older adults’ ability to carry out tasks of daily living, reduce their independency and increase the risk of sarcopenia (4, 5).

Major health organizations, including the World Health Organization (WHO), have emphasized the important role of regular physical exercise as a therapeutic tool to delay age-related functional decline and treat functional limitations (6). Therefore, a physically active lifestyle represents a key behavior to maintain physical function by advancing age (7), where engagement in moderate-to-vigorous physical activity (MVPA) is related to better functional health outcomes (8–10). Current PA guidelines for older adults (65+) stipulate engagement in 150–300 weekly minutes of aerobic type PA of at least moderate intensity to promote healthy aging (11). Furthermore, the WHO also indicates that additional health benefits would be achieved by accumulating twice the recommended minimum amount of aerobic type MVPA (300 weekly minutes) (11). Indeed, based on data from two databases the NHS (Nurses’ Health Study) and the HPFS (Health Professionals Follow-Up Study) including 116,221 female nurses with an age range between 30 and 76 years, it was shown that adults reporting at least twice the recommended minimum amount of either vigorous intensity PA or moderate intensity PA exhibited a significantly lower mortality risk compared to those reporting less time in PA of any intensity (12). Interestingly, a recent meta-analysis of observational studies including 36,383 participants (mean age: 62.6 years) based on objective assessment of PA using accelerometry reported that higher PA volume are related to a lower risk for premature mortality irrespective of the intensity level (13). Notably, the dose–response relationship between time in MVPA and all-cause mortality did not reveal any further significant risk reduction beyond a daily average amount of 25 min MVPA, which roughly corresponds to the minimum weekly recommended amount of MVPA, i.e., 150 min. Furthermore, using baseline data from the Alberta Caring for Diabetes (ABCD) cohort study including 1948 participants no further benefits on health-related quality of life were denoted between participants accumulating twice the recommended minimum amount of MVPA and those reporting less MVPA time (14). However, as indicated by the authors of that study the lack of statistical power may provide an explanation to the lack of significant differences between those who met baseline recommendations and those who adhered to twice the minimum recommendation (14). Thus, the proposed additional benefit of exceeding recommended minimum amount of MVPA remains a matter of debate and is likely dependent on the health outcome of interest. Currently, the extent to which additional accumulation of twice the minimum amount of MVPA benefits indicators of physical function in older adults is unknown. This is unfortunate given the importance of maintained physical function during the aging process. Additionally, the exploration of the potential benefits of accumulating MVPA time beyond the minimum recommendations would be substantially strengthened by the use of objectively assessed MVPA time, complemented by self-reported information regarding the type of activities performed. Indeed, in addition to the weekly aerobic type MVPA, older adults should engage in muscle-strengthening activities (MSA) at least twice a week (11). Emerging evidence suggests that engagement in MSA is related to a lower risk of sarcopenia (15), lower fall incidence (16), lower prevalence of obesity (17), and lower mortality risk (18). Therefore, adherence to the MSA guideline should be taken into account when investigating the potential benefits of accumulating twice the recommended minimum amount of aerobic type MVPA on indicators of physical function in older adults.

The aim of the present study was to assess physical function in older adults who accumulate at least 150 min/week but less than 300 min/week of MVPA compared to those accumulating at least 300 min/week, while taking into account engagement in MSA, protein intake and central obesity.

## 2. Materials and methods

### 2.1. Participants

Seventy-one men (67 ± 2 years) and 122 women (67 ± 2 years) were recruited through local advertisement. Inclusion criteria included the following: adherence to the recommended weekly amount of at least 150 min of MVPA including absence of overt diseases, cardiovascular, diabetes and psychiatric conditions and disability issues regarding immobility. All procedures were conducted according to the principles set by the Declaration of Helsinki and all participants were provided with written information regarding the study and written signed consent was obtained. The study protocol was approved by the regional ethics committee of Uppsala, Sweden (Dnr, 2017/511).

### 2.2. Anthropometry

All anthropometric measurements were performed by trained personal. Body weight and body height were measured using standardized conditions and following standard procedures (19). Participants arrived at the testing facility between 8:00 a.m. and 9:30 a.m. after an overnight fast. Weight was recorded using a Tanita scale (Tanita MC-780, Tanita Amsterdam, The Netherlands) to the nearest 0.1 kg. Body height was measured using a portable stadiometer (Seca 213, Hamburg, Germany) to the nearest 0.1 cm. Waist circumference (WC) was measured to the nearest 0.1 cm at the midpoint between the iliac crest and lower costal margin in a standing position (19) using a measuring tape (Seca 201, Hamburg, Germany).

### 2.3. Physical activity

Daily time spent in MVPA was assessed using the Actigraph GT3x (Actigraph, Pensacola, FL, United States) accelerometer, as previously described (20). All participants were instructed to wear the activity monitor during awake time for seven consecutive days with exception for showering or other water-based activities. The activity monitor was placed at the right hip using an adjustable waste-mounted elastic belt. Participants were instructed not to alter their daily living routines during the measurement period. A minimum of 10 h of daily wear time accumulated during at least 4 days was required for inclusion in further data analysis of physical activity. Accelerometer count cut-point for MVPA was set to >2019 counts per minute according to Troiano et al. (21). Based on average daily time spent in MVPA, participants were classified as physically active (accumulating ≥150 min but <300 min of MVPA per week) or as highly physically active (accumulating ≥300 min of MVPA per week). Engagement in MSA was assessed using the EPAQ2 questionnaire, which has previously been validated (22). Accordingly, participants reported on duration and frequency of MSA during the last 12 months regarding the following activities: strength training, yoga and qigong types, rhythmic gymnastics, rubber band resistance exercises, water-based gym, group-based workout, DVD-based resistance exercises, core workout, and sit-ups. Participants reporting MSA at least twice a week were classified as adhering to the MSA guideline.

### 2.4. Protein intake

Daily protein intake was assessed by a validated food frequency questionnaire (FFQ) (23). Daily protein intake was expressed as g protein/kg body weight. Participants with a daily protein intake of at least 1.1 g/BW were classified as meeting recommendations on adequate protein intake in older adults (24).

### 2.5. Physical function

Handgrip strength (expressed in kg/kg body weight) was assessed by standardized procedures using a Jamar handheld dynamometer (Patterson Medical, Warrenville, IL, United States). Handgrip strength was assessed in standing position with elbow flexed 90° and wrist in neutral position. Participants performed 3 maximal attempts separated by 1.5 min of rest, where the highest score was recorded. A five times sit-to-stand test (5-STS; expressed in seconds, s) was performed, whereby participants were instructed to start from a seated position in a chair to a fully upright standing position and to sit down back in the chair. This sequence was repeated five times. The participants were also instructed to place their arms across their chest during the test and to perform this sequence as quickly as possible. Squat jump performance (expressed in N/kg body weight) was assessed on a force platform (Kistler 9,281 B, Kistler Nordic AB, Sweden). Participants started from a static position with knee bent in a 90° angle, with their hands kept on the hip during the jump. Participants performed 3 maximal jumps separated by 1.5 min of rest. The highest recorded maximal ground reaction force during the concentric phase of the squat jump was recorded (25). A six-minute walk test (6MWT, expressed in meters, m) (26) was used to assess functional walking capacity and cardiorespiratory fitness. Participants were instructed to walk as fast as possible along a 50-meter corridor for a period of 6 min. Participants were allowed to rest in case of any discomfort during the 6 MWT. The total distance walked was recorded.

### 2.6. Physical performance score

A continuous clustered physical performance score (PPS) covering the separate physical function indicators was created. The PPS was derived by first transforming each physical function indicator into sex-specific standardized units (z-scores). Thereafter, the four standardized variables were summed and averaged into one sex-adjusted composite variable, with a mean value of zero and where a positive score indicates a better physical function compared to a negative score. Since longer time to complete the 5STS test indicates a lower physical function, data on 5STS were inverted before creating the PPS.

### 2.7. Statistical analyses

Data are presented as mean and standard deviation unless otherwise stated. Data were checked for normality using the Kolmogorov–Smirnov normality test and visual inspection. In case of deviations from normality, data were log-transformed to better fit a normal distribution. Differences in physical function indicators between the physically active and highly physically active groups were analyzed using a factorial analysis of variance (ANOVA). All models were adjusted by sex, engagement in MSA and adherence to protein intake guideline. In addition, models analyzing 6MWT and 5-STS performances were further adjusted by waist circumference to account for adiposity level on weight-related exercises. *A priori* power calculation showed that small to moderate effect sizes were detected with a power of ≥80%, when based on our sample size and alpha level set to 0.05. All analyses were conducted using SPSS version 28 (SPSS, Chicago, IL, United States).

## 3. Results

A total of 193 community-dwelling older men (*n* = 71, 67 ± 2 years), and women (*n* = 122, 67 ± 2 years) were included in the analysis. Forty-eight percent of the participants (*n* = 93) accumulated ≥150 but <300 weekly minutes of MVPA and 52% (*n* = 100) accumulated ≥300 weekly minutes of MVPA. Correspondingly, 27% (*n* = 52) of the participants engaged in MSA for at least twice a week. Thirty-three percent of the participants (*n* = 63) adhered to the protein recommendation of at least 1.1 g/kg/day. Data on anthropometric measurements in physically active and highly physically active older men and women are presented in Table 1. Data on indicators of physical function in the two groups are presented in Table 2.

**Table 1:** Body composition variables in the study sample.

	Physically active		Highly physically active	
	Men (*n* = 35)	Women (*n* = 58)	Men (*n* = 36)	Women (*n* = 64)
Height (cm)	177.0 ± 5.3	164.3 ± 5.9	179.2 ± 6.4	165.6 ± 4.6
Weight (kg)	80.4 ± 12.9	64.6 ± 9.1	79.5 ± 8.9	63.0 ± 9.2
Waist circumference (cm)	95.1 ± 11.1	79.6 ± 8.2	92.3 ± 8.6	78.7 ± 8.9

Data are presented as mean ± SD.

**Table 2:** Indicators of physical performance in the study sample.

	Physically active (*n* = 93)	Highly physically active (*n* = 100)
Squat Jump (N/kg bw)	9.4 ± 2.3	9.8 ± 2.2
Handgrip (kg/kg bw)	0.48 ± 0.1	0.50 ± 0.1
5-STS (s)	10.3 ± 2.0	10.0 ± 2.3
6MWT (m)	631 ± 63	655 ± 62^*^

Data are presented as mean ± SD. 5-STS, 5-time sit to stand; 6MWT, Six-minute walk test; kg bw, kilogram body weight. ^*^p < 0.05. vs. Physically active.

**Figure 1:**
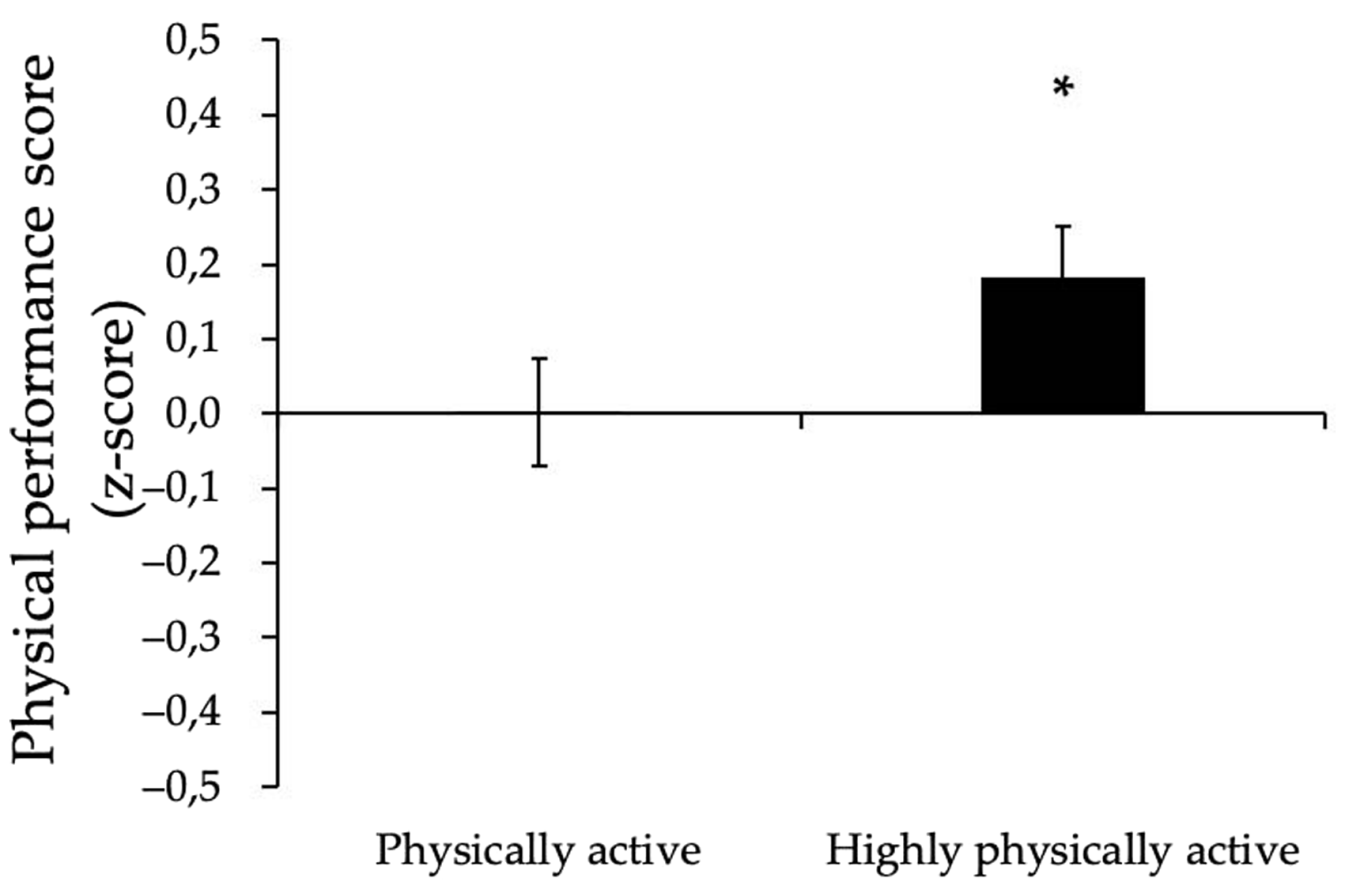
Physical performance score (z-score) in physically active and highly physically active older adults. Data are means ± SEM, adjusted for MSA, sex, waist circumference and protein intake. ^*^p < 0.05.

We first compared clustered PPS between the physically active and highly physically active groups. Factorial ANOVA revealed a significantly higher PPS in the highly physically active compared to physically active group (0.12 ± 0.07 vs. −0.13 ± 0.07, *p* < 0.05). Importantly, further adjustments by MSA, sex, WC and protein intake did not change the significance of the results (Figure 1).

Further analysis of each separate indicators of physical function showed that the highly physically active group had a significantly better 6MWT performance (*p* < 0.05) compared to the less active group (Table 2), which remained evident after adjustment for MSA, sex, WC, and protein intake (664 ± 6.09 m vs. 644 ± 6.46 m, *p* = 0.01). In contrast, no significant group differences in squat jump, hand grip strength and 5-STS were observed (Table 2).

## 4. Discussion

The World Health Organization recommends older adults to engage in a minimum of 150 weekly minutes of aerobic type PA of at least moderate intensity, while also advocating the potential health benefits of moving beyond this minimum threshold toward a doubling of the weakly recommended amount of aerobic type MVPA (300 weekly minutes) (11). The scarcity of data addressing potential benefits of accumulating twice the minimum amount of MVPA on health outcomes in older adults may be explained by a generally low PA level in age groups similar to ours, i.e., above 65 years. Moreover, the benefits of doubling the minimum recommended PA amount on indicators of physical function in aging populations have not been previously explored. In the present study we show for the first time that accumulation of ≥300 weekly minutes of MVPA is related to a significantly better physical function, which is driven by a significantly better walking performance in older adults when compared to older adults who accumulate between 150 and 300 min/week.

A recently published large prospective US cohort study showed that adults reporting at least twice the recommended minimum amount of MVPA exhibited a significantly lower mortality risk compared to those with less MVPA time (12). Interestingly, the authors of this study including 116,000 participants aged between 30 and 76 years showed that engaging in twice the amount of either moderate or vigorous intensity PA would result in similar health benefits. In our study, times spent in moderate and vigorous intensity PA were not analyzed separately, hence the impact of PA intensities above the moderate threshold on physical performance outcomes cannot be clarified. Notably, none of the participants in our study accumulated twice the recommended amount of weekly vigorous PA (150 min or more). Therefore, data on PA spent in moderate and vigorous PA were collapsed into one MVPA variable showing that aerobic-type activities of at least moderate intensity, such as brisk walking, is beneficial for cardiorespiratory fitness in older adults, as mirrored by the better 6MWT performance.

The results from our current study expand on the findings from Lee et al. (12) showing that benefits of being physically active beyond the minimum recommended MVPA level also include a better physical function. However, in contrast to the favorable impact on walking performance, no corresponding benefits were observed for any indicator of muscle strength and function. Handgrip strength, a proxy for overall muscle strength commonly used for strength assessment in older adults (27) was similar in the two groups. It may be hypothesized that most activities performed within the MVPA domain were predominantly of aerobic type, which would unlikely elicit significant improvements in forearm muscle flexors (28). Further, although the 5-STS is an established functional test for assessment of functional ability and lower limb strength (29), it may lack sensitivity to detect differences between participants who already adhere to the MVPA guideline. However, we further took advantage of using an instrument with higher sensitivity, a force platform, to determine lower limb strength, which confirmed the lack of beneficial impact of accumulating twice the minimum recommended MVPA amount on lower limb strength. However, we have previously reported (15) that engagement in MSA alongside adherence to the minimum weekly MVPA time is positively related to indicators of muscle strength and function. Thus, it can be speculated that doubling the recommended aerobic type MVPA amount alone would not be sufficient to infer benefits on all dimensions of physical function including muscle strength.

The fact that adherence to the MSA guideline was considered in the statistical analysis strengthens the finding that adherence to twice the recommended MVPA amount is related to better walking performance. Importantly, adherence to MSA guideline should still be encouraged alongside adherence to the aerobic-type guideline for optimal impacts on physical function during aging, as MSA can improve important fall risk factors including strength and flexibility (30). These findings may hold important clinical and public health implications on the ability to perform activities of daily living and reducing the burden of physical disability and related health-care costs during the aging process.

Importantly, the findings from our study are strengthened by the use of both objectively assessed PA and self-reported engagement in MSA. Further, important factors known to impact on physical performance, including sex, protein intake, adiposity level and physical activity type (MSA) were considered in the analysis. It should be noted that for the specific purpose of the present study, only physically active older adults were included, which implies that data on indicators of physical function may not be representative of a larger sample of older adults with sedentary lifestyles. Finally, due to the cross-sectional analysis, causality cannot be determined.

In conclusion, the present study shows that accumulating at least 300 weekly minutes of MVPA is linked to a better walking performance in older adults compared to those just adhering to the minimum weekly amount of MVPA. However, in order to promote maintenance of muscle strength and function, engagement in MSA is likely necessary alongside adherence to the aerobic-type MVPA guideline.

## Data availability statement

The raw data supporting the conclusions of this article will be made available by the authors, without undue reservation.

## Ethics statement

The studies involving human participants were reviewed and approved by the Regional Ethics Committee of Uppsala, Sweden (Dnr, 2017/511). The patients/participants provided their written informed consent to participate in this study.

## Author contributions

JV, FK, and AN: conceptualization. JV, PE, FK, and AN: methodology. JV: formal analysis and writing—original draft preparation. JV, PE, LR-Z, MF, FK, and AN: investigation and writing—review and editing. FK, PE, and AN: supervision. FK and AN: project administration. FK: funding acquisition. All authors read and agreed to the published version of the manuscript.

## Funding

This research was funded by the EU HORIZON 2020 Research and Innovation Program (Euro-pean Joint Programming Initiative “A healthy diet for a healthy life” “JPI HDHL,” the ERA-NET co-fund HDHL-INTIMIC) GA no. 727565 and The Kamprad Family Foundation for Entrepre-neurship, Research and Charity (ref 20210070).

## Conflict of interest

The authors declare that the research was conducted in the absence of any commercial or financial relationships that could be construed as a potential conflict of interest.

## Publisher’s note

All claims expressed in this article are solely those of the authors and do not necessarily represent those of their affiliated organizations, or those of the publisher, the editors and the reviewers. Any product that may be evaluated in this article, or claim that may be made by its manufacturer, is not guaranteed or endorsed by the publisher.
